# The Relationship Between Zinc and Quality of Life in Patients With Upper GI Cancer on Chemotherapy

**Published:** 2017-05-01

**Authors:** Edith A. Brutcher, Zhengjia Chen, Anqi Pan, Tiffany Barrett

**Affiliations:** 1 Department of Hematology and Oncology, Winship Cancer Institute of Emory University, Atlanta, Georgia;; 2 Department of Biostatistics, Winship Cancer Institute of Emory University, Atlanta, Georgia

## Abstract

This is a pilot study aimed at evaluating the prevalence of zinc deficiency and how zinc levels affect the quality of life (QOL) of patients with upper gastrointestinal (GI) cancers receiving systemic chemotherapy. The data collection was completed on 40 patients. Although the primary objective of a positive prevalence of zinc deficiency in upper GI cancer patients at diagnosis and after receiving chemotherapy is not statistically significant, we found a statistically significant association between zinc level and certain QOL factors. There is a significantly positive association with satisfaction of social contact at baseline only, sexual pleasure at baseline and at 2 months, QOL at baseline only, and troublesome sweating at baseline, and from baseline to 2 months corresponding with change in other skin problems. Conversely, there is a significantly negative association corresponding changes in enjoyment of physical activities, how the patient usually feels, sexual pleasure, the way in which the patient approaches food, QOL, rashes on the face, and other skin problems. Neutropenia grades were reflective of decreased zinc at baseline but did not show decreased zinc correlating with a weakened immune system.

Malnutrition is commonly observed in patients with cancer. This prevalence could be even higher depending on the location and stage of the tumor. The highest prevalence is found in those patients with tumors of the gastrointestinal (GI) tract and the lungs (Inui, 2002). At least 80% of patients who present with stomach or pancreatic cancer have or develop malnutrition (Bosaeus, Daneryd, Svanberg, & Lundholm, 2001).

Multiple factors contribute to the malnutrition associated with cancer, and they include poor appetite, treatment-related nausea, and malabsorption due to enteritis from chemotherapy. In addition to these factors, patients with gastric and pancreatobiliary cancers have an impaired digestion due to involvement of the GI tract with cancer. Therefore, malnutrition is very common in this group of patients. A common sign of malnutrition in patients with cancer is hypoalbuminemia (Scheinfeld, Lin, & Mokashi, 2011). Identification of two or more of the following six criteria is recommended for diagnosis of malnutrition: insufficient energy intake, weight loss, loss of muscle mass, loss of subcutaneous fat, localized fluid accumulation, or diminished functional strength (White, Guenter, Jensen, Malone, & Schofield, 2012).

## THE ROLE ZINC PLAYS IN DISEASE

Protein malnutrition correlates with inadequate intake of many essential micronutrients such as zinc (Scheinfeld et al., 2011). Zinc is bound to albumin; therefore, hypoalbuminemia can result in lower serum zinc levels (Jen & Yan, 2010). Zinc plays important roles in growth and development, immunity, neurologic function, and reproduction. Zinc is absorbed in the small intestine by a carrier-mediated mechanism. Generally, 33% is accepted as the average zinc absorption in humans. Zinc absorption has a positive relationship with the amount of protein in a meal (Lönnerdal, 2000). Zinc deficiency has been linked with alopecia and nail dystrophy (Yu, Yang, Shan, & Lin, 2011), as well as effects of the central nervous, immune, and skeletal systems (Tuerk & Fazel, 2009).

Zinc plays a role in the body’s response to stress. Under major stress, people tend to lose zinc in urine, sweat, and saliva. Low zinc levels can be found in people with depression, and low zinc levels can affect inflammation and immunity.

The highest amount of zinc in the body is found in the brain, but the body has no special zinc-storage capability (Deans, 2013). The brain- derived neurotrophic factor (BDNF) is a key factor in the development of depression. The activity of zinc in the brain, in part, is to stimulate the production of brain-derived neurotrophic factor (Patenaude, 2013).

Zinc-containing neurons throughout the hippocampus subserve mood regulation and cognitive functions (Swardfager et al., 2013). Zinc is one of the micronutrients involved in behavior, learning, and mental functions (Ranjbar et al., 2013). Animal studies showed zinc replacement reduced symptoms of depression and anxiety (Ramer, 2015), and animal models showed zinc treatment had antidepressant-like effects (Swardfager et al., 2013). Zinc supplements together with selective serotonin-reuptake inhibitor antidepressants improved responses in patients with major depressive disorders (Ranjbar et al., 2013). Low zinc levels, therefore, may be a biomarker for depression (Deans, 2013).

The incidence of zinc deficiency in patients with upper GI cancers is unknown, as is the impact of chemotherapy on the prevalence of zinc deficiency. Since the symptoms of decreased zinc levels overlap with several of the symptoms of chemotherapy, it is difficult to estimate the impact of zinc levels on the quality of life (QOL) and toxicity profile of patients with upper GI cancers receiving chemotherapy.

## STUDY DESIGN

We hypothesized that patients with upper GI cancers have a higher incidence of decreased zinc levels and zinc deficiency associated with hypoalbuminemia. Our secondary objectives were to correlate serum zinc levels with symptoms such as fatigue; depression; rash; dry, itchy skin; neutropenia; and hypoalbuminemia. Therefore, the incidence of decreased zinc levels after administration of chemotherapy may impact the patients’ QOL and their recovery from side effects of chemotherapy.

To test these hypotheses, we conducted a pilot study to evaluate the prevalence of zinc in newly diagnosed patients with upper GI malignancies prior to treatment and while on chemotherapy at 2 and 4 months. After signing consent with the primary investigator, patients had bloodwork drawn at three key time points (prior to treatment, 2 months on treatment, and 4 months on treatment); they also completed QOL and self-reported skin complaints surveys at the time of the blood draws. The two valid QOL questionnaires used in this trial are the Moorehead-Ardelt Quality of Life Questionnaire: Self-Esteem and Activities (Moorehead, Ardelt-Gattinger, Lechner, & Oria, 2003) and the Dalgard Self-Reported Skin Complaints survey (Dalgard, Svensson, Holm, & Sundby, 2003). The data were collected and reviewed for correlations relative to the hypotheses.

**Patient Selection**

This study was approved by the institutional review board at Emory University. Patients were considered eligible for the trial if they had a diagnosis of previously untreated nonresectable gastric, gastroesophageal, pancreatic, or biliary cancer. Prior therapy in the adjuvant or neoadjuvant setting was not allowed. Patients with any surgical intervention in the setting of gastric, gastroesophageal, pancreatic, or biliary cancers were also ineligible.

Blood for zinc levels and albumin were collected at baseline, defined as within 2 weeks of starting chemotherapy (Table 1). Repeat measurements of zinc and albumin levels were performed at 2 and 4 months. Complete blood cell counts were performed as per standard of care, which was usually every other week. Quality of life was assessed using the two questionnaires previously mentioned. The surveys included questions such as the following: How do you feel about yourself? Are you satisfied with your social contacts? How do you approach food? Do you derive pleasure from sex? Have you experienced dry rash, itchy skin, or other skin problems? The QOL survey was completed by the patients at baseline as well as at 2 and 4 months.

**Table 1:**
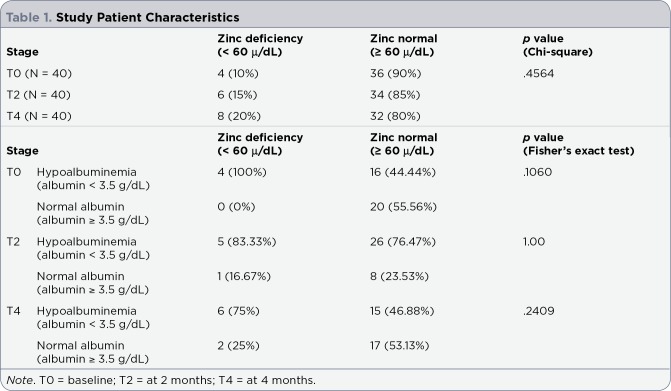
Study Patient Characteristics

**Endpoint Definition and Sample Size**

Zinc was considered within normal limits if it was at or above the standard institutional laboratory parameters of 60 µg/dL. Serum albumin was defined as within normal limits if it was at or above the institutional laboratory parameters of 3.5 g/dL. Neutropenia was graded as per Common Terminology Criteria for Adverse Events (CTCAE), version 4.0.

This is a pilot study aimed at evaluating the relationship between malabsorption, zinc, and QOL in patients with upper GI cancers on chemotherapy. Patients are considered eligible if they completed 4 months of therapy. The null hypothesis is there will be no significant positive outcome on the QOL of patients with upper GI cancers in correlation with zinc levels.

The sample size was based on 80% power to detect a difference of 0.4 between the null hypothesis and the alternate hypothesis of 0.6, with an estimated standard deviation of 1.0 and a significance level of 0.05 using a one-sided one sample t-test. Based on that estimate, 40 patients will be evaluable for the primary endpoint prevalence of zinc deficiency or changes in zinc levels at diagnosis and after 4 months of chemotherapy in patients with cancers of the upper GI system.

**Statistical Analysis**

Univariate associations between categorical variables were examined with chi-squared test or Fisher’s exact test, where appropriate. Univariate associations between continuous variables were examined with both Spearman and Pearson correlation. Analyses were performed using SAS 9.4 (SAS Institute, Inc., Cary, North Carolina) with two-sided tests and a significance level of 0.05.

## RESULTS

For the 40 patients with completed data, demographic characteristics follow: 20 (50%) were male, 21 (52%) were white, 13 (32%) were black, 1 (2%) was Asian, and 5 (12%) were other. Median age was 60 years (range, 39–81 years).

Review of the data showed no significant difference in the prevalence of zinc deficiency among the 3 time points of prior to treatment, at 2 months, and at 4 months into treatment (Table 2). There was no significant difference in the prevalence of hypoalbuminemia between patients with zinc deficiency and those without at each time point. There was a significantly positive association between the zinc value and the albumin level at baseline. There was no significant correlation between any changes in zinc and albumin. There was a significantly negative association between the zinc value and the neutropenia grades at baseline only. There were no significant correlations between any changes in zinc and neutropenia grades.

**Table 2:**
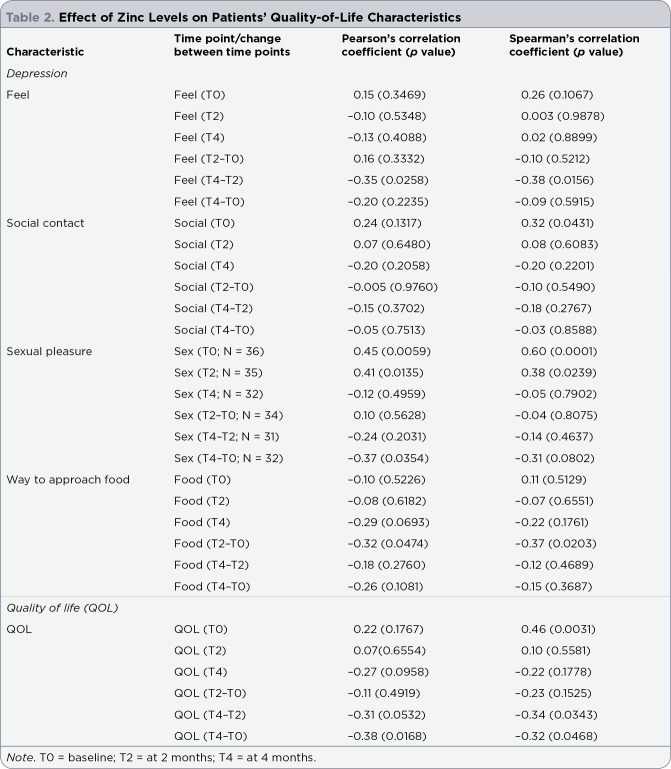
Effect of Zinc Levels on Patients’ Quality-of-Life Characteristics

**Table 2:**
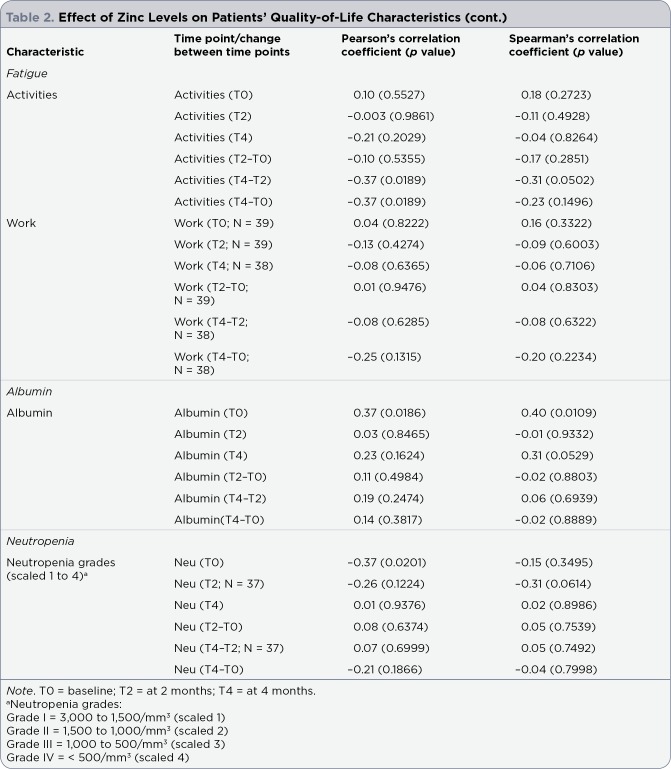
Effect of Zinc Levels on Patients’ Quality-of-Life Characteristics (cont.)

There was no significant correlation between zinc value and fatigue. The data did, however, find changes of zinc levels from baseline to 4 months and from 2 months to 4 months significantly negatively associated with the corresponding change in the feeling of whether the patient enjoyed physical activities. There was no significant correlation between zinc value and how the patient usually feels at each time point. Changes in zinc levels from 2 months to 4 months were significantly negatively associated with the corresponding change in how the patient usually feels.

The data showed the zinc value was significantly positively associated with satisfaction of social contact at baseline. There was no significant correlation between any changes in zinc levels and in the satisfaction of social contact. The zinc value was significantly positively associated with sexual pleasure at baseline and at the 2 months. The changes in zinc levels from baseline to 4 months were significantly negatively associated with the corresponding change in sexual pleasure.

There was no significant correlation between the zinc value and the way in which a patient responded on the QOL survey related to how they approach food at any time point. Changes in the zinc level from baseline to 2 months were significantly negatively associated with the way in which the patient responded to how they approached food on the QOL survey. The zinc value was significantly positively associated with the QOL at baseline only. Changes in zinc levels from baseline to 4 months and from 2 months to 4 months were significantly negatively associated with the corresponding change in QOL.

There was no significant correlation between zinc value at each time point and no significant correlation between any changes in zinc value and corresponding change in itchy skin, dry/sore rash, scaly skin, itchy rash on hands, pimples, warts, and loss of hair. There was no significant correlation between zinc value and other rashes on the face at each time point, respectively. Changes in zinc levels from 2 months to 4 months were significantly negatively associated with the corresponding change in other rashes on the face.

The zinc value was significantly positively associated with troublesome sweating at baseline. There was no significant correlation between any changes in zinc value and troublesome sweating. There was no significant correlation between zinc value and other skin problems at each time point. Changes in zinc levels from baseline to 2 months were significantly positively associated with the corresponding change in other skin problems.

## DISCUSSION

It is estimated that one-third of the global population is deficient in zinc. This in part is related to underdeveloped countries surviving on food that is inadequate with zinc content and with infections that generally cause zinc to be sequestered in the liver (Stein, Quaim, & Nestel, 2007). Symptoms of zinc deficiency include poor neurologic function, weak immunity, increased sensitivity to allergic reactions, hair thinning, and acne or rashes (Axe, 2015). Studies suggest that people with low zinc levels are at greater risk of developing conditions of the aging process (Pepersack et al., 2001). Zinc levels decline with aging, and zinc supplementation has been shown to stabilize cognition in those with Alzheimer’s disease (Brewer, 2012). In addition, patients with major depressive disorder have been found to have lower zinc levels (Swardfager et al., 2013). In a study with children, it was found that higher serum zinc levels correlated with decreased levels of anxiety and depression (Ramer, 2015).

Patients with cancer may have micronutrient deficiency for a variety of reasons. Micronutrients are often insufficient at the time of diagnosis, and their levels may deteriorate after starting therapy for cancer. In addition to inadequate intake and side effects of treatment, inflammatory response can affect concentrations of certain vitamins and minerals (Maryland, 2004).

This pilot study did not identify a statistically significant positive prevalence of zinc deficiency and corresponding low albumin level in patients with gastric and pancreatobiliary cancers at diagnosis or after receiving chemotherapy. There was also no significant correlation between any changes in zinc levels and neutropenia grades or albumin levels. There were however, some associations between zinc levels and QOL. There was a statistically significant association found in relationship to changes in zinc levels and QOL.

Looking at the correlations with QOL issues and zinc values, there were a few positive results. There was a significantly positive association with zinc value and QOL along with the patient’s satisfaction with social contact at baseline. The zinc value was also significantly positively associated with the patient’s sexual pleasure at baseline and at 2 months. Conversely, there was a significantly negatively associated correspondence with neutropenia grades at baseline only.

When we looked at the data related to changes in zinc levels prior to starting chemotherapy and at 2 and 4 months after starting chemotherapy, there was a significant negative association with the corresponding change in the feeling of whether a patient enjoys physical activities and in how a patient usually feels. Changes in zinc levels were significantly negatively associated with the corresponding change in a patient’s sexual pleasure and significantly negatively associated with the way in which a patient approaches food. Therefore, when the zinc level decreased, the patient’s enjoyment of sex and food decreased. The data show that changes in the zinc level from baseline to 4 months and from 2 months to 4 months are significantly negatively associated with QOL. Again, when the zinc level decreased, the patient’s feelings about QOL decreased.

We hypothesized that patients with pancreatobiliary and gastric cancers have a high incidence of zinc deficiency. We also hypothesized that the incidence of zinc deficiency increased after administration of chemotherapy and that zinc deficiency impacts QOL and recovery from the side effects of chemotherapy. Although the primary objective was not met, and the data did not support the incidence of zinc deficiency in patients with gastric, gastroesophageal, pancreatic, or biliary cancers, the data in this pilot study did support the effect of changes in zinc values on QOL. The data from the patients being treated for cancer did show a correlation between zinc level changes during treatment and how they perceived their QOL.

Patients on chemotherapy experience distress, anxiety, and depression (Pandey et al., 2006). Looking forward from this study, with data supporting changes in zinc levels having an impact on patients with upper GI cancer receiving systemic chemotherapy and their QOL, a follow-up study would help to evaluate the impact of zinc replacement on the identified QOL issues. The goal is to improve our patients’ QOL by defining and delineating the benefit of preventing changes in zinc level throughout treatment with systemic chemotherapy. A follow-up study to evaluate the impact of zinc replacement on the identified QOL issues in the same population of patients could also evaluate the benefit of monitoring and maintaining zinc levels in these patients.

## CONCLUSION

Although patients with upper GI cancers have many challenges, the indications from this study are that patients’ QOL may be improved with prevention of zinc deficiency and decreases in zinc levels throughout systemic chemotherapy. Proper assessment of nutritional intake and treatment of nausea, vomiting, and diarrhea may help prevent zinc deficiency and changes in serum zinc levels. A follow-up of this pilot study will further evaluate the impact of zinc levels on patients’ QOL, including enjoyment of physical activities, emotional state, sexual pleasure, dietary habits, and skin changes.

**Acknowledgments**

The authors would like to acknowledge the Emory Nell Hodgson Woodruff School of Nursing, and Dr. Bassel El-Rayes in the preparation of this article.
